# A Neuroeconomic Theory of Mental Time Travel

**DOI:** 10.3389/fnins.2018.00658

**Published:** 2018-09-26

**Authors:** Isabelle Brocas, Juan D. Carrillo

**Affiliations:** ^1^Department of Economics, University of Southern California, Los Angeles, CA, United States; ^2^CEPR, London, United Kingdom

**Keywords:** declarative memory, mental time travel, neuroeconomic theory, memory-based process, attention

## Abstract

We propose a theoretical model that places *attention* at the center of mental time travel (MTT) ability. This theory predicts that attention promotes a memory-based process that encodes memories of unexpected events, facilitates accurate recollection of information of such events during MTT, and optimizes subsequent decision-making. This process coexists with a habitual process that governs all other events and treats them equally. Our theory demonstrates that the memory-based process is useful when the environment features novel experiences that are likely to be relevant in future decision-making, hence worth remembering accurately. By contrast, the habitual process is optimal in environments that either do not change significantly, or have a small chance of being repeated in the future. This may explain why the ability to mentally travel in time has developed differently in humans than in other species. Implications are discussed in the context of decision-making.

## 1. Introduction

According to the classic Tulving theory (Tulving, 1985a), memory has the distinctive mission of permitting humans to mentally travel backwards and forwards in time. Mental time travel (MTT) allows us to recall past events to avoid dangers and choose the best future courses of action. Central to MTT is episodic memory, which processes memories of personal events and experiences and makes it possible to use past experiences to simulate future events or alternative pasts. Episodic memory summarizes sensory, perceptual, and emotional information and features a visual representation component. It is part of the declarative memory system, which encodes precise information that can be verbalized, but also easily forgotten (Squire, 2004; Poldrack and Foerde, 2008)^1^. The relationship between episodic memory components and MTT has been widely documented. From a developmental perspective, MTT develops as soon as children understand the concept of “yesterday” and “tomorrow,” and in conjunction with the development of episodic memory (Suddendorf and Busby, 2005; Hayne and Imuta, 2011). The MTT ability is also less developed in subjects with a poor visual imaginary or those who tend to suppress emotions (D'Argembeau and Van der Linden, 2006).

The neural correlates of episodic memory are well-understood. Episodic memory involves the medial temporal lobe (MTL), which hosts the hippocampus. Dysfunction of the latter (amnesia) has been associated with either no memory of personal events or a difficulty in forming new memories about personal events further associated with no ability to project oneself in the future (Tulving, 1985b; Klein et al., 2002; Hassabis et al., 2007). The MTL is also implicated in episodic future thinking –or the construct of future possible scenarii– and episodic counterfactual thinking –or the simulation of alternative pasts– (Schacter et al., 2015). Interestingly, episodic memory has been shown to interact with working memory (Balconi, 2013). In particular, increased activation of the dorsolateral prefrontal cortex (dlPFC) during the formation of episodic memories results in their enhancement. Working memory also plays a role in the episodic buffer, which allows the use of old memories to form new ones. The dlPFC has been shown to have a causal role in episodic memory formation and to be responsible for suppressing memories through suppressing hippocampal processing (Benoit et al., 2014). These findings taken together indicate that a neural circuit involving hippocampal regions and working memory regions are at the core of episodic memory. As such, we conjecture that an interplay between these structures affects the selection of memories worth being recalled in the future.

MTT is often believed to be uniquely human and to have been shaped through evolution (Suddendorf et al., 2009). However, episodic memory capacity depends on a fundamental neural circuit that is similar across mammalian and avian species, which suggests a shared underlying neural ancestry rather than a specific human evolutionary trajectory (Allen and Fortin, 2013). It is therefore still unclear to which extent this capacity supports MTT in animals (Suddendorf and Busby, 2003; Suddendorf and Corballis, 2007). Working memory capacity is also believed to have evolved differentially across species, and humans are thought to be unique in some of the uses they make of it (Carruthers, 2013). These differences in both MTT and working memory capacities between humans and non-humans may be related. Fitness depends on the environments that species encounter, and some environments require specific abilities to adapt. In theory, an environment requiring better prospective and processing capabilities may have triggered the evolution of modern human working memory and MTT abilities. Hence, the specific interplay between episodic memory and working memory might have been shaped by evolution differentially across species to adapt to different environments.

These observations taken together suggest that MTT ability is supported by episodic memory, which is involved in an interplay with the working memory system to encode memories of relevance as a function of the environment. Said differently, observations are compatible with the existence of a goal-directed (top down) memory management mechanism that allocates attentional resources to form memories with the objective of maximizing future rewards. In this article, we consider a stochastic environment that produces events relevant for decision-making. These can be memorized and later recalled to make decisions. We build a theoretical framework to identify the most efficient interplay between a system capable of forming memories at a cost (episodic memory) and a goal-directed mechanism that allocates attentional resources in order to best serve future decision-making in a given environment.

To identify such an efficient mechanism, we adopt an optimization approach. This approach is widely used in decision-making fields such as Economics, and has been applied to model behavior driven by interactions between brain systems (Bernheim and Rangel, 2004; Brocas and Carrillo, 2008, 2014). This approach consists of endowing brain systems (e.g., those involved in goal-directed attention) with the ability to optimize their behavior (e.g., allocate attention) conditional on the stimulus they receive (e.g., features of an event) and the goal to fulfill (e.g., the relevance of the event in future decisions)^2^. This approach captures two critical features of brain processing. First, it recognizes brain modularity and the fact that each system is handling a specific task. Second, it envisions systems as being tuned to operate efficiently to interpret the information they receive and to formulate a response.

## 2. Remembering the past to predict the future

### 2.1. The basic model

We consider a mathematical formulation of MTT, which extends and complements the simple two-memory system representation introduced by Brocas and Carrillo (2016). We use a deliberately simplistic car-parking example to illustrate this theory. First, an event occurs. It corresponds to the location in the parking lot where the individual (from now on “he") has parked in the morning. We denote this event by *x*. The event can be conceptualized as a draw from the random variable *X* which, in our example, is simply the distribution of parking spots that the person has been using in the past. We assume that *X* follows a normal distribution:

$$X\;\sim \;N\left(\theta ,\frac{1}{p}\right).$$

This means that the person usually parks around the spot θ. The parameter *p*, the precision (or inverse of the variance) of the random variable *X*, captures how much the draw varies from day-to-day. In our example, it represents how congested the parking lot is, and therefore how likely it is to find a spot near θ. By abuse of language we will refer to θ, the average of the distribution, as a “typical” realization of the event.

The individual needs to form a memory about the event. He recruits the episodic memory for this purpose. In our example, the memory represents the individual's recollection of the spot where he parked his car in the morning. To model imperfect recollection of events, we assume that when the individual travels back in time at a later date, his memory *m* of the true event *x* is given by:

(1) $$m=x+u\;where\;u\;\sim \;N\left(0,\frac{1}{e}\right).$$

The memory is a distorted representation of the true event. The size of the distortion is inversely related to the amount of attentional resources *e* invested when forming the memory. More precisely, the distortion takes the form of a noise *u* that follows a normal distribution with mean 0 and precision *e*. In expectation, the memory is correct, that is, there is no systematic bias in the distortion: *E*(*m*) = *x*. Under infinite attention (*e* = +∞), the individual will perfectly recall the state (*m* = *x* with certainty), and with no attention (*e* = 0), the recollection is infinitely vague. Given attentional resources are scarce, there is an opportunity cost of allocating them to this task. We assume that the cost of attention is increasing and convex, formally represented by a quadratic cost function *c*(*e*) = *e*^2^/2. Thus, the main difference relative to Brocas and Carrillo (2016) is that, in the present paper, the individual chooses between a continuum of possible levels of attention (*e* ∈ [0, +∞)) rather than only two levels (*e*_1_ and *e*_2_ with *e*_2_ > *e*_1_ > 0).

The recollected memory *m* is used to plan an action *a*. In the context of our example, the action of the individual is where to look for his car when it is time to go home. The individual obtains a payoff inversely related to the distance between the true location and the location where the individual looks. Again for simplicity, we model this payoff with a standard quadratic utility loss function:

$$-l{\left(a-x\right)}^{2}$$

with *l* > 0. According to this formulation, if the event (true location) *x* is recalled with exactitude (*m* = *x*), the individual's optimal action is:

$$\tilde{a}(x)=\text{arg }\underset{a}{\text{max}}\;-l{\left(a-x\right)}^{2}\;\Rightarrow \;\tilde{a}(x)=x.$$

**Figure 1:**
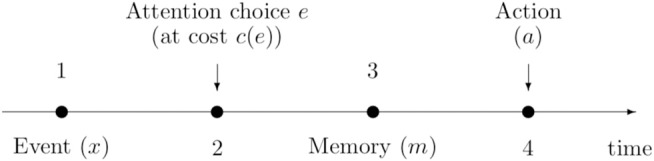
Timing of the decision making problem.

This simply means that it is optimal to look for the car wherever it is located. Deviations from ã(*x*) –looking for the car in a different spot– implies a loss (e.g., a time delay or a longer walk) which increases with the absolute distance |*a* − *x*| and the sensitivity of the individual to losses (*l*). The timeline of the decision process is summarized in Figure 1.

This individual decision problem has standard multi-stage features. It is formally solved by backward induction.

### 2.2. Optimal action given available memory

If a finite amount of attention *e* has been allocated to the task, the true location will not be recalled with exactitude. Instead an (imperfect) memory *m* will be retrieved. Given that memory, the action that maximizes the expected payoff is:

$$a^{*}(m,e)=\text{arg }\underset{a}{\text{max}}\;-\int_{x}l{\left(a-x\right)}^{2}dF_{e}(x|m)$$

where *F*_*e*_(*x*|*m*) is the revised cumulative distribution function of the event *x* given the attention exerted (*e*), and the memory (*m*). Given a quadratic objective function, the optimal decision satisfies the first-order condition and is given by:

(2) $$a^{*}(m,e)=E_{e}[X|m],\;$$

where E_*e*_ is the expectation operator. The optimal action coincides with the expected belief about the event given the memory retrieved. The normality assumption of the variables *X* and *u* implies that:

(3) $$m|x\;\sim \;N\left(x,\frac{1}{e}\right)\text{ and }X|m\;\sim \;N\left(\frac{p}{e+p}\theta +\frac{e}{e+p}m,\frac{1}{e+p}\right)$$

According to (3), the memory is centered around the true event *x* but with a distortion that depends inversely on the attentional effort *e*. Once a memory is retrieved, the updated belief about the event (*X*|*m*) is a random variable. Its expected value is a convex combination of the prior θ and the memory *m*. Combining Equations (2, 3), we can rewrite the optimal action as:

(4) $$a^{*}(m,e)=\frac{p}{e+p}\theta +\frac{e}{e+p}m$$

Given limited attention, memories are known to be imperfect and therefore cannot be fully trusted. Hence, in our example, it is optimal for the individual to look for his car not where his memory tells him to (*m*) but, instead, somewhere between the typical location and where his memory tells him to (between θ and *m*). If more attention has been dedicated to memorize the event (*e* high), the memory is more reliable, so the action is closer to the memory. The action is also closer to the memory when the dispersion in choices is higher (*p* low) since, in that case, the knowledge about the typical location is less valuable. This is summarized as follows (proofs of all Propositions can be found in the Supplementary Material section).

Proposition 1. *Suboptimal actions are the result of imperfect memories and they are modulated by attention. Individuals can trust their memories more if they are highly attentive (e high) and if their typical behavior is less reliable (p low)*.

### 2.3. Optimal attention anticipating the future action

We now use backward induction to determine the optimal attention e^*^ in stage 2 given the anticipated optimal action a^*^ to be exerted in stage 4 as determined in Equation (4) (see Figure 1).

After observing the event *x*, allocating attention *e* affects the distribution from which memories are drawn, which we denote by *G*_*e*_(*m*|*x*). Given a memory *m*, the action will subsequently be $a^{*}\left(m,e\right)={\text{E}}_{e}\left[X|m\right]$. Therefore, the expected payoff of allocating attention *e*, net of opportunity costs, is:

$$\begin{array}{l} V_{x}(e)=-\int_{m}l{\left(a^{*}\left(m,e\right)\;-x\right)}^{2}dG_{e}(m|x)-\frac{e^{2}}{2}\\ \;=-l(\frac{e+p^{2}{\left(x-\theta \right)}^{2}}{{\left(e+p\right)}^{2}})-\frac{e^{2}}{2}. \end{array}\;$$

It is optimal to allocate resources that achieve the highest expected payoff, namely:

$$e^{*}=\text{arg }\underset{e}{\text{max}}\;V_{x}(e),$$

which leads to the following conclusion:

Proposition 2. *There exists a range of events* [*x*, *x*] containing the typical event (*x* < *θ* < *x*) such that it is optimal not to exert any attention (e^*^ = 0) when the realization of the event falls in that range. Some level of attention is optimal (e^*^ > 0) *when the realization falls outside that range* (*x* ∉ [*x*, *x*]). *In that case, attention increases as the event moves away from the typical realization*.

The idea behind this result is simple. The event is a stochastic variable centered around θ. When the current realization of the event is sufficiently close to this value (|*x* − θ| small), it is not worth remembering. Instead it is a better strategy to save on attentional resources, act as if the location is θ and incur a moderate loss −*l*(*x* − θ)^2^. This case corresponds to a form of “habitual thinking,” that is a thinking process based on general prior information rather than accurate information. By contrast, when the realization is sufficiently far from it (|*x* − θ| large), it is worth exerting attention to remember the event with precision and avoid an action excessively far from the optimal one. In our example, if the individual has parked the car near the typical spot θ, it is worth not paying any attention and looking in that spot first, but if the individual has parked very far from it, then it is worth remembering the location with some accuracy.

Overall, when *x* is observed, the individual exerts an optimal level of attention e^*^ and the average action he undertakes is given by:

(5) $$E[a^{*}|x]=\{\begin{array}{ll} \theta & \text{if}x\in [\underline{x},\bar{x}]\\ \;\frac{p}{e^{*}+p}\;\theta +\frac{e^{*}}{e^{*}+p}x\; & \text{if}x\notin [\underline{x},\bar{x}] \end{array}$$

**Figure 2:**
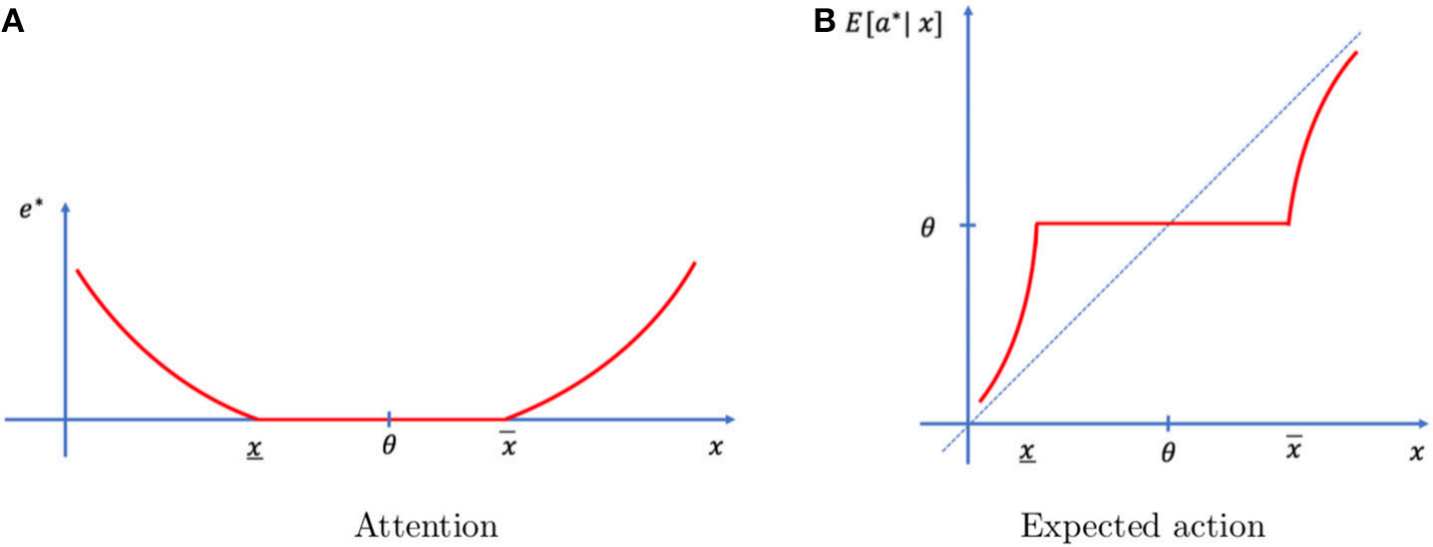
Optimal mechanism. **(A)** No attention is allocated (e^*^ = 0) when the event is close to typical (*x* close to θ) and attention increases as the event becomes more unexpected (*x* far from θ). **(B)** In the absence of memory limitations, actions should follow the 45 degree line. Under memory limitations, actions are based on prior knowledge (a^*^ = θ) when no attention has been allocated (*x* ∈ [*x*, *x*]) and it is based on the memory signal when attention has been allocated. As events are more extreme, memory signals are less distorted, and actions are closer to optimal.

The optimal solution of the problem is summarized in Figure 2. The individual exerts more attention when the realized event is far from typical (Figure 2A) resulting in an action that is more congruent with the event (Figure 2B). Events that are close to typical (*x* ∈ [*x*, *x*]) do not require any attention to be memorized and they are followed by typical actions (θ).

### 2.4. Modulating attention and choice

Attention and actions are modulated by features of the environment. We can perform comparative statics to measure how changes in specific features of the problem affect the amount of attention exerted and the subsequent action undertaken.

#### 2.4.1. The effect of stakes

**Figure 3:**
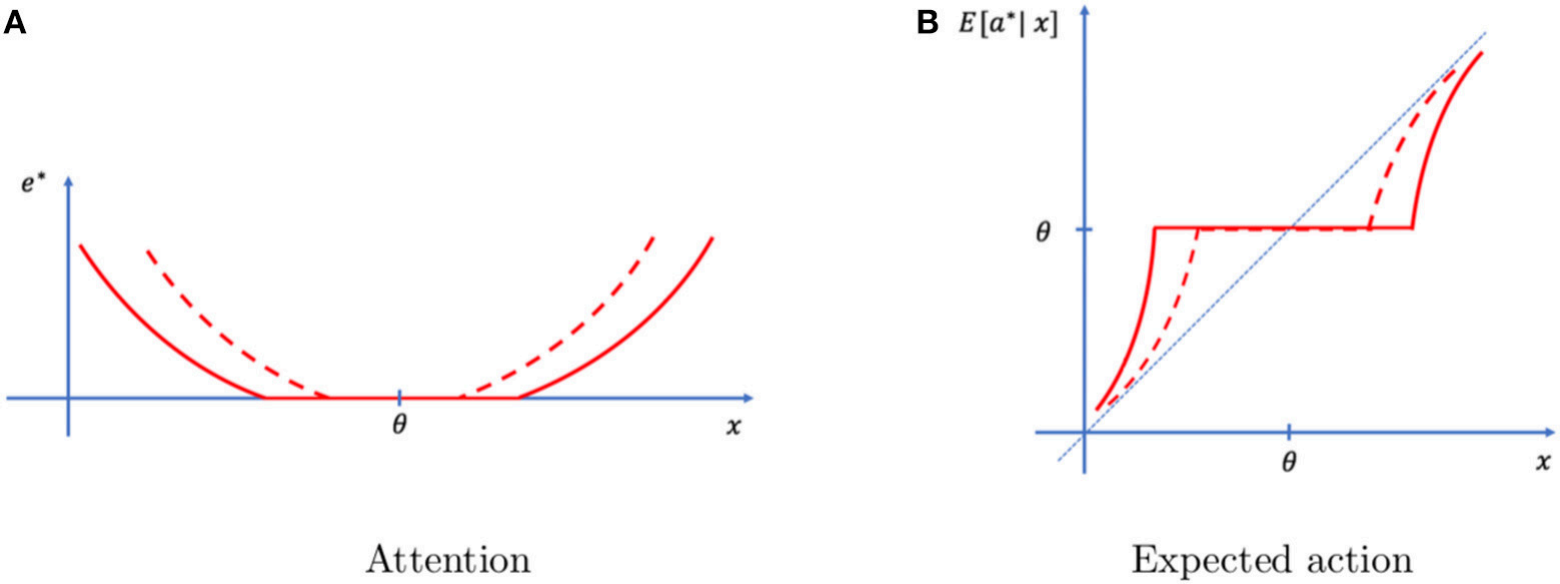
Effect of stake. Optimal attention **(A)** and decisions **(B)** under low stakes (full line) and high stakes (dashed lines). As stakes increase, attention increases and actions are on average closer to the event. Also, the range of events for which no attention is exerted shrinks.

Stakes are captured in our setting with the parameter *l*, that measures the sensitivity to losses. Not surprisingly, the optimal level of attention is increasing in the sensitivity to losses. If the individual expects to incur a large disutility of not finding his car quickly, he should allocate more attention to the formation of a reliable memory. This in turn impacts the decision. With a higher sensitivity to losses comes greater attention, and therefore more reliable memories. These memories are trusted more, which is reflected in actions closer to the memory and farther away from the typical event. We represent this effect in Figure 3.

Note that *l* can equally capture the likelihood that the information contained in the event will be useful in the future. Under this interpretation, our theory predicts that it is efficient to invest more attention when this likelihood increases.

Proposition 3. *As stakes increase, attention increases, making memories more precise and reliable. As a result, actions rely more heavily on the memory and less on the typical event*.

#### 2.4.2. The effect of the environment

We can compute the expected payoff of the individual before the realization of the event and anticipating that he will allocate an optimal amount of attention. It is given by:

$$\begin{array}{l} EV=\int_{X}-l\left(\frac{e^{*}+p^{2}{\left(X-\theta \right)}^{2}}{{\left(e^{*}+p\right)}^{2}}\right)dF(X)-\frac{{\left(e^{*}\right)}^{2}}{2}\\ \;=-l\left(\frac{1}{e^{*}+p}\right)-\frac{{\left(e^{*}\right)}^{2}}{2}.\; \end{array}$$

In terms of our example, it represents the expected utility of the individual when he is going to work and has not yet parked his car. Interestingly, the individual is better-off when the distribution from which the event occurs is concentrated around its mean (high *p*) or, in our example, when he frequently finds a parking spot close to the typical location. This means that, even though the individual optimally reacts to extreme events by exerting more attention, he still benefits when such events do not occur frequently. An environment that changes little offers a guarantee to obtain safe rewards at minimal cost.

Proposition 4. *Environments in which events do not vary much (high p) are conducive of habitual thinking and yield high expected rewards*.

Notice that by choosing attention over a continuum of options, we can determine the marginal effect of stakes and events on the optimal level of attention. Perhaps more importantly, this model delivers new results relative to Brocas and Carrillo (2016). In particular, we can show that no attention is sometimes optimal and that the individual is better-off when the environment does not change significantly. These results have behavioral and neurophysiological implications that are discussed in the next section.

## 3. Implications of the theory

According to our theory, decision-making that requires knowledge of previous events is optimized through an interplay between the episodic memory system (with capabilities to form memories) and the attentional system (with capabilities to enhance memories). This interplay is orchestrated by goal-directed mechanisms that select relevant memories and *optimize* their collection to best serve decision-making. This overall process makes it possible to recall past events with more or less accuracy, and to simulate future rewards informed by past memories. Importantly, our model also shows that memories and behavior resulting from this process are modulated by a series of parameters describing the environment in which decisions are made.

### 3.1. Dual process theories

The central result of the theoretical section is the existence of two regimes, one in which events are not memorized and decisions are based on prior information and another in which events are memorized and (imperfectly) recollected to optimize decision-making. As such, our theory provides support for the coexistence of a habitual process (which operates in the limit, when attentional resources are too difficult to be recruited or not worth the cost) and a memory-based process (which is modulated by the amount of attentional resources allocated to the task). These correspond to events that fall inside and outside the range [*x*, *x*], respectively (see Figure 2).

The habitual process is optimal when events are close to typical (*x* close to θ), and when decisions are not very important (*l* small). It is an efficient process associated with simple strategies that rely on prior information about the environment. However, whenever stakes increase or events are rare, the costly memory-based process should be activated to help integrate the important specific features into decisions. This theoretical finding is consistent with the existing literature. Indeed, behavioral studies have suggested that we often use heuristics that rely on limited information about past events (Bröder and Schiffer, 2003, 2006). Recent neuroimaging analyses have also shown that processing more precise information requires a memory-based process that taxes regions involved in working memory, such as dlPFC (Khader et al., 2011, 2016). Our theory rationalizes these findings and identifies the conditions under which heuristic-based decision-making (which does not require precise memory or the ability to simulate the future based on the past) is efficient and when it should be replaced by a costly memory-based process. Moreover, the theory predicts that structures involved in working memory should be recruited when events are far from typical (or unexpected), which is consistent with evidence implicating the dlPFC in these types of events (Kapur et al., 1997; Fletcher et al., 2001).

### 3.2. Dysfunctions

Our theory can be extended to address the behavioral implications of dysfunctions of the two main systems involved in memory formation, namely working memory and episodic memory.

#### 3.2.1. Working memory dysfunction

Critical to our results is the working memory system that makes it possible to form and recall episodic memories of relevance for decision making. In the context of our model, the disorder can be captured by rescaling the cost function. More precisely, the cost of attention can vary across individuals and be given by *c*(*e*) = α*e*^2^/2 where α represents the idiosyncratic cost of a unit of attention (in the basic model, α = 1). This modification affects the cost-benefit trade-off reported in section 2.4. Using the same methodology as in section 2, we notice that the optimal allocation of attention is decreasing in α (see the Appendix in **Supplementary Material** for the formal derivation). Said differently, this theory predicts that dysfunctions of the working memory system result in the allocation of fewer resources to remember events. As the cost of attention increases, the region [*x*, *x*] becomes larger. This, in turn, implies that the habitual process becomes more prevalent and decisions are more often based on the typical realization of events rather than on accurate information. Interestingly, working memory disorders (such as attention deficit hyperactivity disorders) have been associated with episodic memory deficits in particular in complex memory tasks (Felton et al., 1987; Quinlan and Brown, 2003). Working memory disorders generally relate to the inability to allocate attention to the task at hand or for the duration required to complete it. Individuals act as if allocating attentional resources is extremely costly and decision-making trade-offs turn to favor limited attention. This description is consistent with the predictions of the model: when working memory becomes taxing, it is optimal to rely on habitual processes.

#### 3.2.2. Episodic memory dysfunction

Mental time travel abilities have been shown to not work properly in amnesic patients, who can neither remember episodic memories nor simulate future events (Klein et al., 2002). Amnesia refers to the inability to encode memories and is an extreme case of an episodic memory dysfunction. Such dysfunction can be accommodated in our model by assuming that after exerting an amount of attention *e*, the memory is actually encoded with probability *q* and it is not encoded with probability 1 − *q*. Our basic model would correspond to *q* = 1, whereas the case of an amnesic patient incapable of forming any memory would correspond to *q* = 0. This limited ability to form memories affects the optimal allocation of attention as well as subsequent decisions. Indeed, individuals who form memories only very rarely (low *q*) should exercise less attention because the latter is likely to be wasted. Said differently, the theory predicts that dysfunctions of the episodic memory system result in allocating fewer resources to remembering events. As the probability of actually encoding memories decreases, the region [*x*, *x*] becomes larger. Dysfunctions of episodic memories also yield choices closer to heuristic decision-making. This, as in the case of a working memory dysfunction, translates into decisions that rely heavily on the habitual process. In the limit case in which no memory can be formed (*q* = 0), the individual is not able to simulate past events to make decisions and relies exclusively on the habitual system ([*x*, *x*] takes the entire support).

In sum, dysfunctions of the working memory system and the episodic memory system will result in an increased difficulty to form new memories and simulate future events based on these memories. It will also imply an increased tendency to resort to the habitual memory system. Conditional on the dysfunction, this is the best response of the individual to his environment. However, from the perspective of an outside observer, behavior will appear to not reflect the true information contained in the events and to rely on inadequate heuristic rules. This prediction is consistent with the evidence reported in the case of clinical populations, in particular for patients suffering from amnesia (Klein et al., 2002), stress disorders (Brown et al., 2014), schizophrenia (Aleman et al., 1999) and other related dysfunctions of hippocampal regions. Similar memory disorders are also observed as a consequence of normal aging (Friedman, 2013) or neurodegenerative diseases such as Alzheimer's, Parkinson's and Huntington's diseases (Panegyres, 2004), that gradually cause episodic memory dysfunction or executive dysfunctions that affect working memory.

### 3.3. Value-based decision-making

Recent literature in neuroscience has dedicated much attention to value-based decision-making. These decisions involve the choice between options of different values that need to be constructed and compared. This paradigm is used to describe neural correlates of economic choices between items in situations involving time delays, uncertainty or risk. The evidence supports the existence of a common reward system (Levy and Glimcher, 2012). It also shows that decisions may be modulated by activity in regions involved in working memory (such as the dlPFC) in situations requiring more detailed or higher-order information, such as food choices subject to self-control problems (Hare et al., 2009). It is plausible that this activity is related to the retrieval of information encoded in the past and relevant to the current decision. Recent research focuses on the interplay between episodic memory and value based decision-making (Weilbächer and Gluth, 2016), but our understanding of these relationships is still limited. Our theory provides a framework to understand how value is formed at the date of decision as a function of how prior information is encoded. We illustrate these concepts with two examples.

#### 3.3.1. Self-control in the food domain

**Figure 4:**
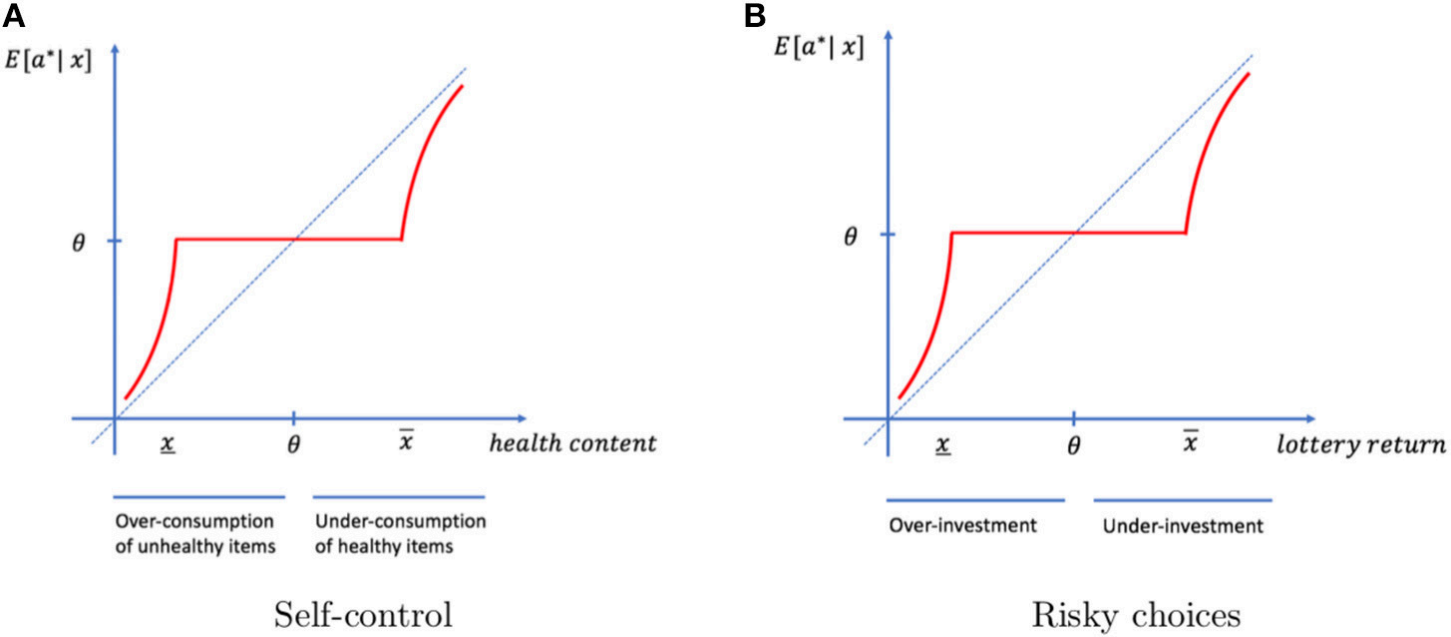
Value-based decisions. **(A)** Items that are less healthy (healthier) than typical will be imperfectly memorized when they are unexpected and not memorized if they are close to typical. In both cases, they are over-consumed (under-consumed) in future episodes. **(B)** Due to imperfect memory, individuals over-invest (under-invest) in lottery tickets they have purchased in the past and turned out to be less (more) profitable than typical.

Let us consider the standard paradigm used in the literature on value and self-control. Suppose that *X* represents the health characteristic of food items. The individual tries food item *x* and learns its health characteristic. This information may be used in a future consumption episode. Knowing the exact characteristic of the food yields optimal consumption decisions. Our model predicts that this information will not be memorized if it is close to typical. The habitual process will feed a habitual value formation and a habitual behavior, while a memory-based process will help the individual choose the behavior that best fits his needs. Furthermore, our theory also implies that the item will be treated as healthier than it really is if *x* < θ, and therefore is over-consumed. Similarly, the item will be treated as less healthy than it really is if *x* > θ, and therefore is under-consumed. Overall and as illustrated in Figure 4A, our theory argues that the efficient memory management process is at the core of biases in behavior.

These biases will be exacerbated if the individual is impulsive and heavily discounts the future (*l* is small) or has a working memory deficit. Interestingly, associations between impulsivity, low discounting and disrupted episodic future thinking have been observed among both healthy and obese subjects (Daniel et al., 2013; Bromberg et al., 2015). Our model is consistent with these findings and suggests some mechanisms. A first possible cause might be an inherent attention deficit that prevents attentional resources to be allocated to the formation of memories. The episodic memory system itself may be intact, but not triggered when necessary. This hypothesis is appealing in the case of eating behavioral disorders, since research in this area has documented disruptions of the working memory system and in particular the dlPFC (Seymour et al., 2015). Another mechanism involves an impaired episodic system that does not form efficient memories and makes the allocation of attentional resources wasteful. Interestingly, eating disorders are also associated with a dysfunction of the mesolimbic regions implicated in processing rewards (Avena and Bocarsly, 2012) and these regions are critical for hippocampal memory formation (Wittmann et al., 2008).

#### 3.3.2. Risk perception

Suppose that *X* represents the expected return of a risky investment (e.g., a lottery ticket). In a given event, the individual purchases a ticket *x* and learns whether it was a good investment. This information may be used in the future to determine whether to purchase it again or not. Our model predicts that lottery tickets associated with returns close to those of typical tickets will be treated equally. In particular, within that region, lottery tickets that have smaller expected returns than typical will be purchased too often (see Figure 4B). Individuals will behave as if they take irrational risks or overestimate their chances of winning. Again, disruptions of the attentional system will exacerbate these inefficiencies. This idea is consistent with studies showing that pathologic gamblers exhibit disruption of this system (Fujimoto et al., 2017). Alternatively, dysfunction of the episodic memory, which is known to be sensitive to reward outcomes, may affect the formation of memories and result in inefficient behavior (Mason et al., 2017).

These results allow us to rationalize both the observed behavior and the neural correlates of behavior that have been reported in the literature. First, the observed interactions between episodic memory regions and attention related regions are a logical response to the need of optimizing memories in order to support decision-making. Second, the behavior resulting from these interactions is efficient. Biases in behavior are the by-product of attentional limitations imposed on information processing.

### 3.4. Time perception biases

The literature on time perception has reported numerous biases on the perception of objective time (Wearden and Lejeune, 2008; Grondin, 2010). For instance, individuals who are told in advance that they will have to make a time judgment later recall time differently than individuals who are not (Block and Zakay, 1997). Time perception can also be manipulated through emotional interventions, such as emotional sounds or pictures, which are likely to disrupt the encoding of relevant time-keeping information (Droit-Volet and Meck, 2007; Fayolle et al., 2015). Indeed, evidence suggests that emotions modulate arousal and attention causing variations in the subjective perception of time (Coull et al., 2004; Droit-Volet and Gil, 2009).

If we reinterpret *x* as the time at which the subject arrived at work this morning and *a* as the report he makes when asked later in the day (θ being his typical arrival time), our model predicts that these reports will vary as a function of the environment. If the individual anticipates that he will need to make a time judgment later and that his accuracy is important (high *l*), the level of attention exerted to memorize the true time will be high, resulting in an accurate memory and a report close to that memory. If he does not anticipate it, he will behave as if the event is not linked to a future reward (low *l*). He will then exert little or no attention and, as a consequence, will report an estimate at or around his usual arrival time. This suggests that the processes involved in retrospective and prospective time paradigms differ and are bound to produce different reports, reflecting implicit manipulations of reward functions.

Along the same lines, if the individual is distracted, overloaded or subject to emotions that impact the amount of attention he can allocate to the task, his behavior can be formally modeled in a similar way to a working memory dysfunction. Our theoretical prediction is that such an individual will be more likely to resort to the habitual system, and will exhibit a less accurate perception of time. Distractions and emotional manipulations will divert attentional resources and prevent the individual from forming correct memories of time. This is again consistent with evidence of increased inaccuracy in the case of mental workload and suggests that attention is central to temporal experience (Brown and Boltz, 2002).

### 3.5. A theory of evolution

If memories are relevant to future choices, then the role of memory is to reconstruct past events to optimize future decisions and collect future rewards. However, if these operations require costly attention, not all events should be memorized and reconstructed equally. Events that are close to typical should not be encoded, since the prior knowledge of the environment is sufficient to guide future decisions with reasonable precision. As events become more striking, different from usual and/or contain important information for future decision-making, it is more important to remember them with accuracy. Overall, the relevance of memories to future rewards should determine the quality of those memories. This strongly suggests that the role of memory and MTT are context dependent. From our analysis, two basic features of the environment are relevant for MTT abilities. First, MTT is necessary when the environment is changing, featuring novel episodes constantly (in our model, large shifts in *x* relative to θ). This suggests that we will resort to costly attention mechanisms to remember rare experiences, but not everyday casual episodes. Hence, we will display the ability to travel back in time to precisely recall these rare experiences. Second, MTT is also necessary when the environment is likely to be relevant in the future (high *l*). Costly attention mechanisms will be recruited for remembering episodes that we would like to repeat in the future, but not events that have no chance of occurring again.

The results obtained with our optimization approach are consistent with evolutionary explanations of the unique time travel human ability (Suddendorf et al., 2009). Given evolution selects features that allow species to adapt to their environment, different evolution patterns should emerge across species as a function of differences in their respective environments. Species that are subject to an environment where the set of events is limited can efficiently operate based on a fixed or heuristic rule, that corresponds to acting as if events were always typical. These decisions do not require goal-directed attention. In such environments, there is no specific need for remembering events, and therefore no specific role for episodic memory or an interplay between episodic memory and attentional processes. This of course does not mean that episodic memory or attention systems have not developed in such species. It simply suggests that they did not need to develop for the purpose of using past events to optimize future decision-making, or that they did not need to develop to the extent humans did.

## 4. Discussion

The objective of our study was to identify the most efficient interplay between a system capable of forming memories (episodic memory) and a goal-directed mechanism that allocates attentional resources in order to best serve future decision-making. We have shown that the optimal way to manage memory formation entails an attention-free habitual system that categorizes events as typical when they are close to what is expected, and a costly memory-based mechanism that optimizes the future recollection of events when they are rare or unexpected. The latter relies on an interplay between brain structures involved in attention and episodic memory regions. The habitual mechanism generates heuristic rules always resulting in the same decision, while the memory-based process allows decisions to be modulated by true events, though imperfectly. This dual system permits an alignment of future decisions with past information in the most efficient way but it results in inefficiencies that depend on the environment. Inefficiencies are larger when events are not predicted to be useful in the future or when relatively rare events are treated as typical.

The conclusion that extreme outcomes are remembered more vividly than typical outcomes (Proposition 2 and Figure 2) relies on the assumption that subjects consciously optimize attention when forming memories. If, in a certain situation, an absent-minded person does not allocate attention as a function of the event, the result would be the opposite, namely a more reliable memory of typical than non-typical outcomes. In our motivating example, such person would be unable to locate the car whenever he parks it in an unusual spot.

Interestingly, and as discussed in section 3, the predictions of the model rationalize existing evidence in terms of both behavior and neural correlates. This has two implications. First, our study supports the idea that the brain processes information efficiently. The existence of different channels (e.g., cognitive or habitual) giving rise to different behavior (e.g., rational or heuristic) relies on a fundamental trade-off between the costs and benefits of information processing. A costly system is triggered when it is worth engaging it. In the context of MTT, our theory suggests that MTT is an invaluable ability when the environment produces events worth remembering. Second, our theory provides a conceptual framework to understand the role of attention in the formation of episodic memories as a function of the environment in which memories are formed. This suggests that the theory can be used to formulate hypotheses in new experimental paradigms. For example, one could design simple memory paradigms that would include a preliminary description of the distribution of experimental events, and would then ask participants to memorize individual events and to later recall them. The main theoretical predictions (Propositions 1 and 2) could be tested by comparing accuracy between expected and unexpected events and by contrasting neural activity across these two types of events. Attentional manipulations could further help assess the causality between attention and accuracy. Moreover, our theory suggests that stakes play a critical role in the formation of memories (Proposition 3). We conjecture that variations in stakes should be associated with differences in neural responses in both episodic memory and working memory regions. This hypothesis could be tested by designing incentivized memory experiments and varying the magnitude of the rewards for remembering accurately. Differences in accuracy should be correlated with differences in activation within regions involved in attention. Last, by comparing the average payoffs of subjects asked to recall similar or different pieces of information over the course of an experiment and by contrasting the patterns of neural activity within attention regions, one could assess whether habitual thinking is more prevalent and results in higher payoffs in environments in which events are more recurrent (Proposition 4). More generally, the model outlined here may be the starting point for a more general, testable neuroeconomic theory of decision-making with imperfect recall.

## Author contributions

All authors listed have made a substantial, direct and intellectual contribution to the work, and approved it for publication.

### Conflict of interest statement

The authors declare that the research was conducted in the absence of any commercial or financial relationships that could be construed as a potential conflict of interest.

## Footnotes

^1^It complements the procedural system that encodes information about skills, an information which is durable but not easy to verbalize.

^2^It is obviously an “as if” approach: even though systems do not intentionally optimize their responses, they are tuned to produce responses that mimic the results of an optimization.
