# Supplementary material for: A Neuroeconomic Theory of Mental Time Travel
Source: Front Neurosci. 2018 Sep 26;12:658. doi: 10.3389/fnins.2018.00658 (PMC6168654; doi:10.3389/fnins.2018.00658)
Supplement: Supplementary file 1 [file Data_Sheet_1.pdf]

Appendix: proofs of Propositions

Proof of Proposition 1

It is immediately implied by equation (4).

Proof of Proposition 2

$$\begin{aligned} V_x(e) &= -\int_m l\,(a^*(m,e)-x)^2\,dG_e(m\,|\,x) - \frac{e^2}{2} \\ &= -\int_m l\left(\frac{p}{e+p}(\theta-x)+\frac{e}{e+p}(m-x)\right)^2 dG_e(m\,|\,x) - \frac{e^2}{2} \end{aligned}$$

Since $m\,|\,x \sim \mathcal{N}\left(x, \frac{1}{e}\right)$ (see equation (3)), the expected payoff can then be rewritten as:

$$\begin{aligned} V_x(e) &= -l\left[\left(\frac{p}{e+p}\right)^2(\theta-x)^2+\frac{2pe}{(e+p)^2}(\theta-x)\mathrm{E}[m-x]+\left(\frac{e}{e+p}\right)^2\mathrm{E}[m-x]^2\right]-\frac{e^2}{2} \\ &= -l\left(\frac{e+p^2\,(x-\theta)^2}{(e+p)^2}\right)-\frac{e^2}{2}. \end{aligned}$$

Taking derivatives, we get:

$$\frac{\partial V_x(e)}{\partial e} = l\frac{e+p(2p(x-\theta)^2-1)}{(e+p)^3}-e$$

Suppose that $(x-\theta)^2 > \frac{1}{2p}$. It means that $\left.\frac{\partial V_x(e)}{\partial e}\right|_{e=0} > 0$. Since $\lim\limits_{e\to+\infty}\frac{\partial V_x(e)}{\partial e} = -\infty$, by continuity there is at least one interior maximum e^* (>0) such that:

$$\left.\frac{\partial V_x(e)}{\partial e}\right|_{e^*} = 0 \quad\Leftrightarrow\quad l\frac{e^*+p(2p(x-\theta)^2-1)}{(e^*+p)^3} = e^*$$

We next prove that the maximum is unique. Taking derivatives once again:

$$\frac{\partial^2 V_x(e)}{\partial e^2} = l\frac{e+p-3(e+p(2p(x-\theta)^2-1))}{(e+p)^4}-1$$

Substituting e^* we therefore get:

$$\left.\frac{\partial^2 V_x(e)}{\partial e^2}\right|_{e^*} = l\frac{1}{(e^*+p)^3}-\frac{3e^*}{e^*+p}-1$$

So, if there are two values $e_2^* > e_1^*$ such that $\left.\frac{\partial V_x(e)}{\partial e}\right|_{e_2^*} = 0$ and $\left.\frac{\partial V_x(e)}{\partial e}\right|_{e_1^*} = 0$, then:

$$\left.\frac{\partial^2 V_x(e)}{\partial e^2}\right|_{e_2^*} = l\frac{1}{(e_2^*+p)^3}-\frac{3e_2^*}{e_2^*+p}-1 < \left.\frac{\partial^2 V_x(e)}{\partial e^2}\right|_{e_1^*} = l\frac{1}{(e_1^*+p)^3}-\frac{3e_1^*}{e_1^*+p}-1$$

In words, the second derivative at e_2^* must be smaller than the second derivative at e_1^*. However, $\left.\frac{\partial V_x(e)}{\partial e}\right|_{e=0} > 0$ means that e_1^* must be a local maximum, that is, $\left.\frac{\partial^2 V_x(e)}{\partial e^2}\right|_{e_1^*} < 0$, which in turn implies that e_2^* cannot be a local minimum and therefore that the interior equilibrium e^* is unique. Overall, for all $(x-\theta)^2 > \frac{1}{2p}$, the optimal level of attention is uniquely defined and given by the first-order condition, equation (6).

Consider now the case $(x-\theta)^2 = \frac{1}{2p}$. The first-order condition becomes:

$$\frac{le^*}{(e^*+p)^3} = e^*$$

This equation has two solutions $e_1^{**} = 0$ and $e_2^{**} = l^{1/3} - p$. It is easy to check that $\frac{\partial V_x(e)}{\partial e} < 0$ for all $e > e_2^{**}$. There are two cases:

- If $l^{1/3} - p < 0$, then the only solution to the problem is $e_1^{**} = 0 = \lim_{(x-\theta)^2 \to \frac{1}{2p}} e_1^*$ and the function $V_x(e)$ is always decreasing in e. For all $(x-\theta)^2 < \frac{1}{2p}$, $\frac{\partial V_x(e)}{\partial e} < 0$. Hence the solution of the problem is the corner solution $e^* = 0$ for all $(x-\theta)^2 < \frac{1}{2p}$.

- If $l^{1/3} - p > 0$, the interior solution of the problem is $e_2^{**} = \lim_{(x-\theta)^2 \to \frac{1}{2p}} e_1^*$. Notice that $V_x(e)$ is decreasing in $(x-\theta)^2$ for all e. Also, $\dfrac{\partial V_x(e)}{\partial e}$ is increasing in $(x-\theta)^2$ for all e. Given these properties, for all $(x-\theta)^2 < \frac{1}{2p}$, there are at most two solutions: $\hat{e}_1 = 0$ and the solution $\hat{e}_2$ to $\left.\frac{\partial V_x(e)}{\partial e}\right|_{\hat{e}_2} = 0$. Last notice that:

$$\frac{\partial V_x(0) - V_x(\hat{e}_2)}{\partial (x-\theta)^2} = -l + l\frac{p^2}{(\hat{e}_2+p)^2} < 0$$

Furthermore, $\lim_{(x-\theta)^2 \to 0} V_x(0) = 0$ and $\lim_{(x-\theta)^2 \to 0} V_x(\hat{e}_2) < 0$. Therefore, there exists a cutoff k^* ($< \frac{1}{2p}$) such that the solution is $\hat{e}_1 = 0$ for all $(x-\theta)^2 < k^*$. The cutoff k^* is solution of $V_x(0) = V_x(\hat{e}_2)$, that is it solves

$$\left(\frac{p^2\, k^*}{p^2}\right) = \left(\frac{\hat{e}_2 + p^2\, k^*}{(\hat{e}_2+p)^2}\right) - \frac{\hat{e}_2^2}{2}$$

where $\hat{e}_2$ solves

$$l\frac{\hat{e}_2 + p(2pk^* - 1)}{(\hat{e}_2+p)^3} = \hat{e}_2$$

To sum up:
- If $l^{1/3} - p < 0$, there exist two values $\underline{x} = \theta - \frac{1}{\sqrt{2p}}$ and $\overline{x} = \theta + \frac{1}{\sqrt{2p}}$ such that the optimal attention e^* solves equation (6) for all $x \notin [\underline{x}, \overline{x}]$, that is, whenever $(x-\theta)^2 > \frac{1}{2p}$ and the optimal attention is $e^* = 0$ for all $x \in [\underline{x}, \overline{x}]$, that is, whenever $(x-\theta)^2 \leq \frac{1}{2p}$.

- If $l^{1/3} - p \geq 0$, there exist two values $\underline{x} = \theta - \sqrt{k^*}$ and $\overline{x} = \theta + \sqrt{k^*}$ such that the optimal attention e^* solves equation (6) for all $x \notin [\underline{x}, \overline{x}]$, that is, whenever $(x-\theta)^2 > k^*$ and the optimal attention is $e^* = 0$ for all $x \in [\underline{x}, \overline{x}]$, that is, whenever $(x-\theta)^2 \leq k^*$.

For the case where optimal attention is strictly positive (and given by (6)), a straightforward differentiation of the first-order condition yields:

$$\left.\frac{\partial^2 V_x(e)}{\partial e^2}\right|_{e^*} \frac{de^*}{d(x-\theta)^2} + \left.\frac{\partial^2 V_x(e)}{\partial e\, \partial (x-\theta)^2}\right|_{e^*} = 0 \quad \Leftrightarrow \quad \frac{de^*}{d(x-\theta)^2} \propto \left.\frac{\partial^2 V_x(e)}{\partial e\, \partial (x-\theta)^2}\right|_{e^*} > 0$$

which means that in the interior equilibrium, attention increases as x moves farther away from θ. This is reflected in Figure 2a.

Finally, from equation (5), it is immediate that for all $x > \overline{x}$, as x increases, e^* increases so $E[a^* \,|\, x]$ is closer to x. Similarly, for all $x < \overline{x}$, as x decreases, e^* increases so $E[a^* \,|\, x]$ is again closer to x. This is reflected in Figure 2b.

Proof of Proposition 3

Differentiation of the first-order condition in the interior optimum (6) yields:

$$\left.\frac{\partial^2 V_x(e)}{\partial e^2}\right|_{e^*} \frac{de^*}{dl} + \left.\frac{\partial^2 V_x(e)}{\partial e\, \partial l}\right|_{e^*} = 0 \quad \Leftrightarrow \quad \frac{de^*}{dl} \propto \left.\frac{\partial^2 V_x(e)}{\partial e\, \partial l}\right|_{e^*} > 0$$

Proof of Proposition 4

Applying the envelope theorem, we can differentiate EV (the expected payoff before the realization of the event) with respect to p. We obtain:

$$\frac{dEV}{dp} = \frac{\partial EV}{\partial p} = l \frac{1}{(e^* + p)^2} > 0$$

Proof of claims in Section 3.2

Working memory dysfunction. Differentiation of the first-order condition in the interior optimum and given a cost of attention $c(e) = \alpha \frac{e^2}{2}$ yields:

$$\left.\frac{\partial^2 V_x(e)}{\partial e^2}\right|_{e^*} \frac{de^*}{d\alpha} + \left.\frac{\partial^2 V_x(e)}{\partial e\, \partial \alpha}\right|_{e^*} = 0 \quad \Leftrightarrow \quad \frac{de^*}{d\alpha} \propto \left.\frac{\partial^2 V_x(e)}{\partial e\, \partial \alpha}\right|_{e^*} < 0$$

Episodic memory dysfunction. For an encoding probability q, the expected payoff becomes:

$$V_x(e) = -l\left(q\frac{e + p^2\,(x-\theta)^2}{(e+p)^2} + (1-q)(x-\theta)^2\right) - \frac{e^2}{2}.$$

Differentiating again the first-order condition in the interior optimum yields:

$$\left.\frac{\partial^2 V_x(e)}{\partial e^2}\right|_{e^*} \frac{de^*}{dq} + \left.\frac{\partial^2 V_x(e)}{\partial e\, \partial q}\right|_{e^*} = 0 \quad \Leftrightarrow \quad \frac{de^*}{dq} \propto \left.\frac{\partial^2 V_x(e)}{\partial e\, \partial q}\right|_{e^*} > 0$$
